# Supplementary figures and images for: Development of a novel prediction model based on protein structure for identifying RPE65-associated inherited retinal disease (IRDs) of missense variants
Source: PeerJ. 2023 Aug 2;11:e15702. doi: 10.7717/peerj.15702 (PMC10404030; doi:10.7717/peerj.15702)

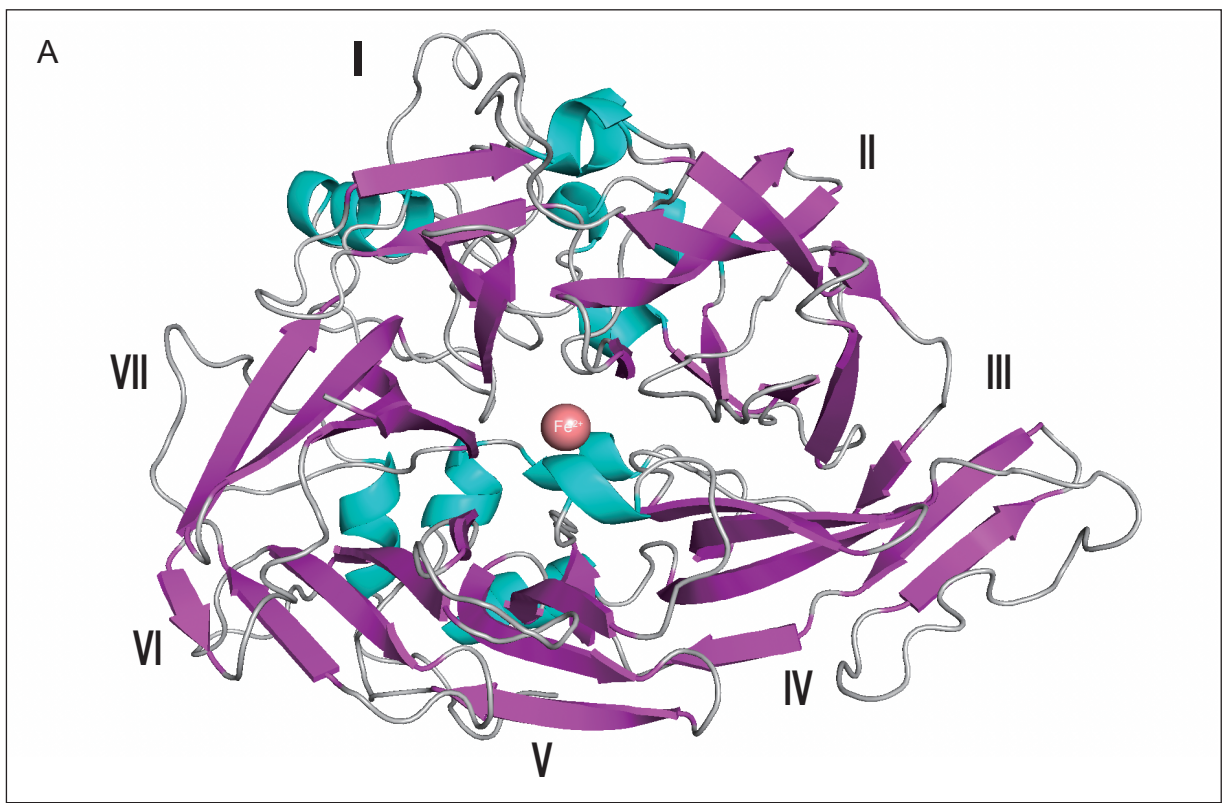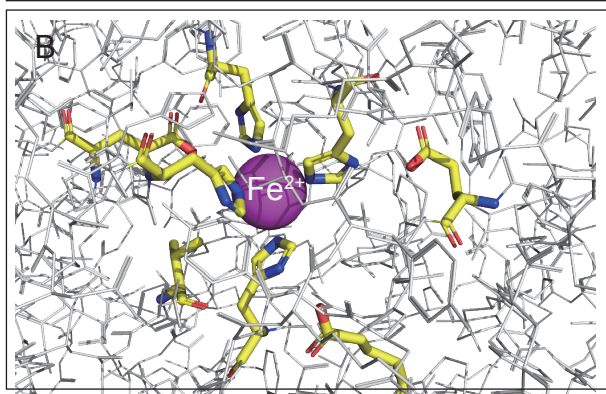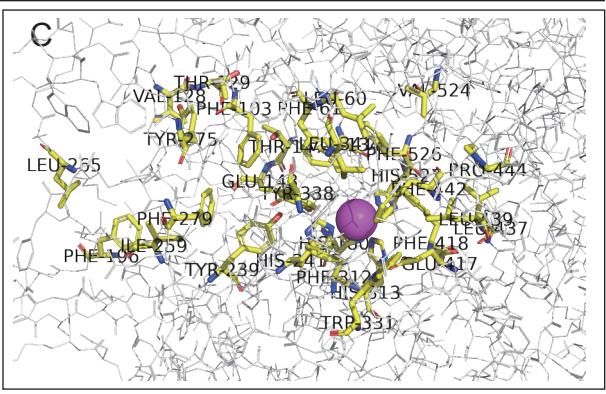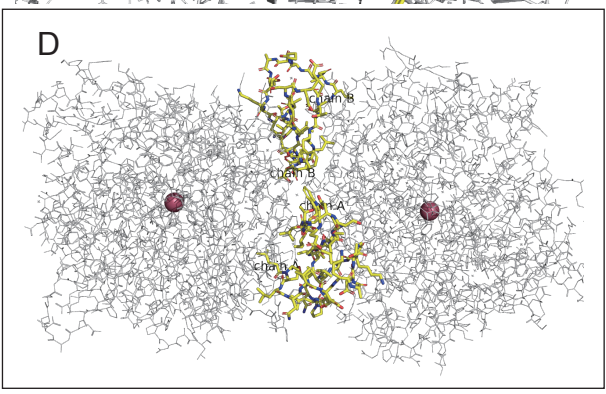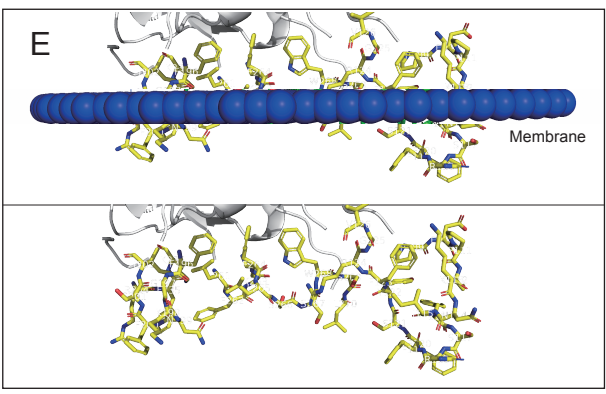

Supplement: Figure S1 — (A) The RPE65 protein is divided into three parts: blue helix, purple sheet, and other structures (gray). (B) The structure of the RPE65 iron center is coordinated by a 4-His/3-Glu motif (148Glu, 180 His, 241 His, 313 His, 417Glu, 469Glu, 527His). (C) Detailed view of the active site cavity. (D) The structure of the RPE65 dimer. (E) Detailed view of the membrane binding residues. [file peerj-11-15702-s001.pdf]

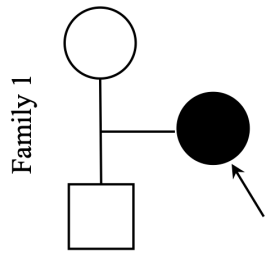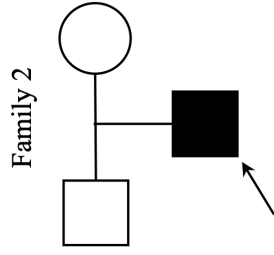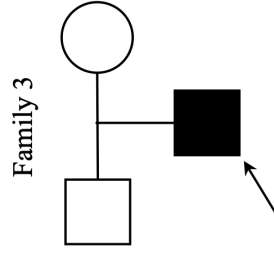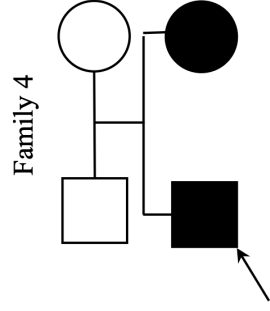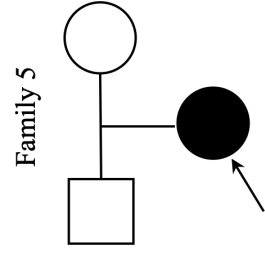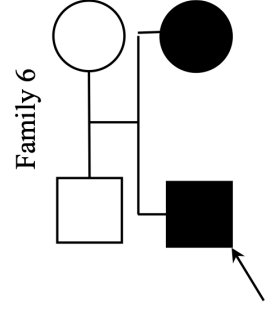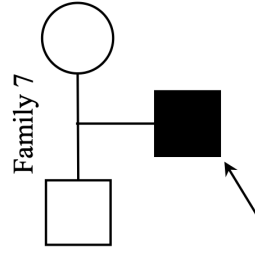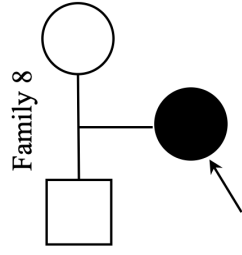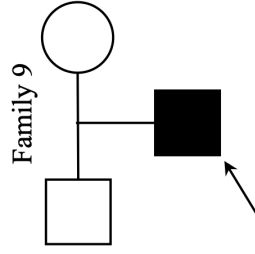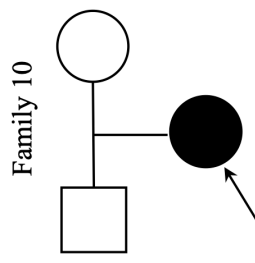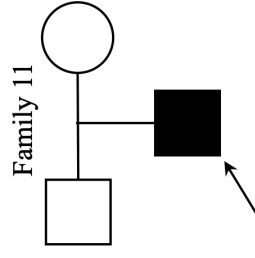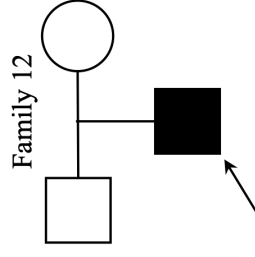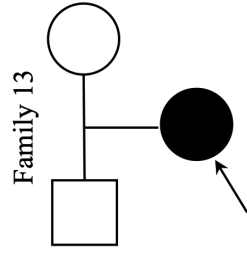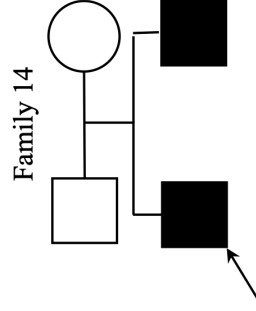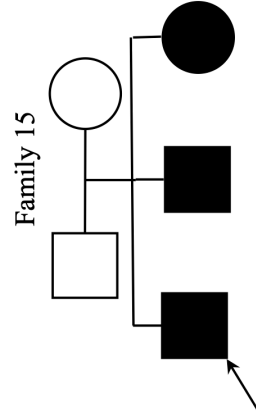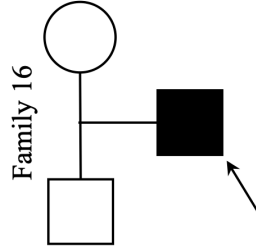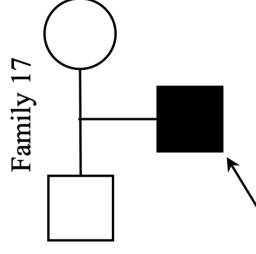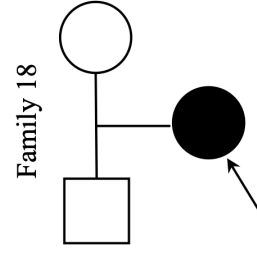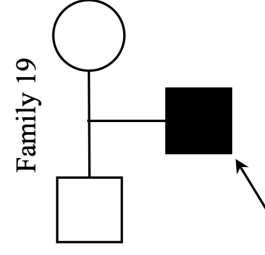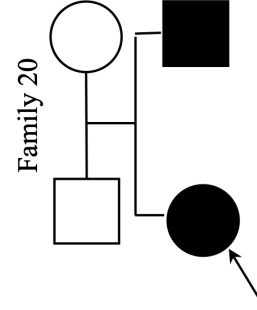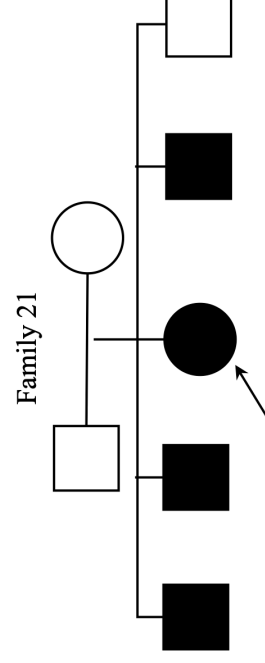

Supplement: Figure S2 [file peerj-11-15702-s002.pdf]

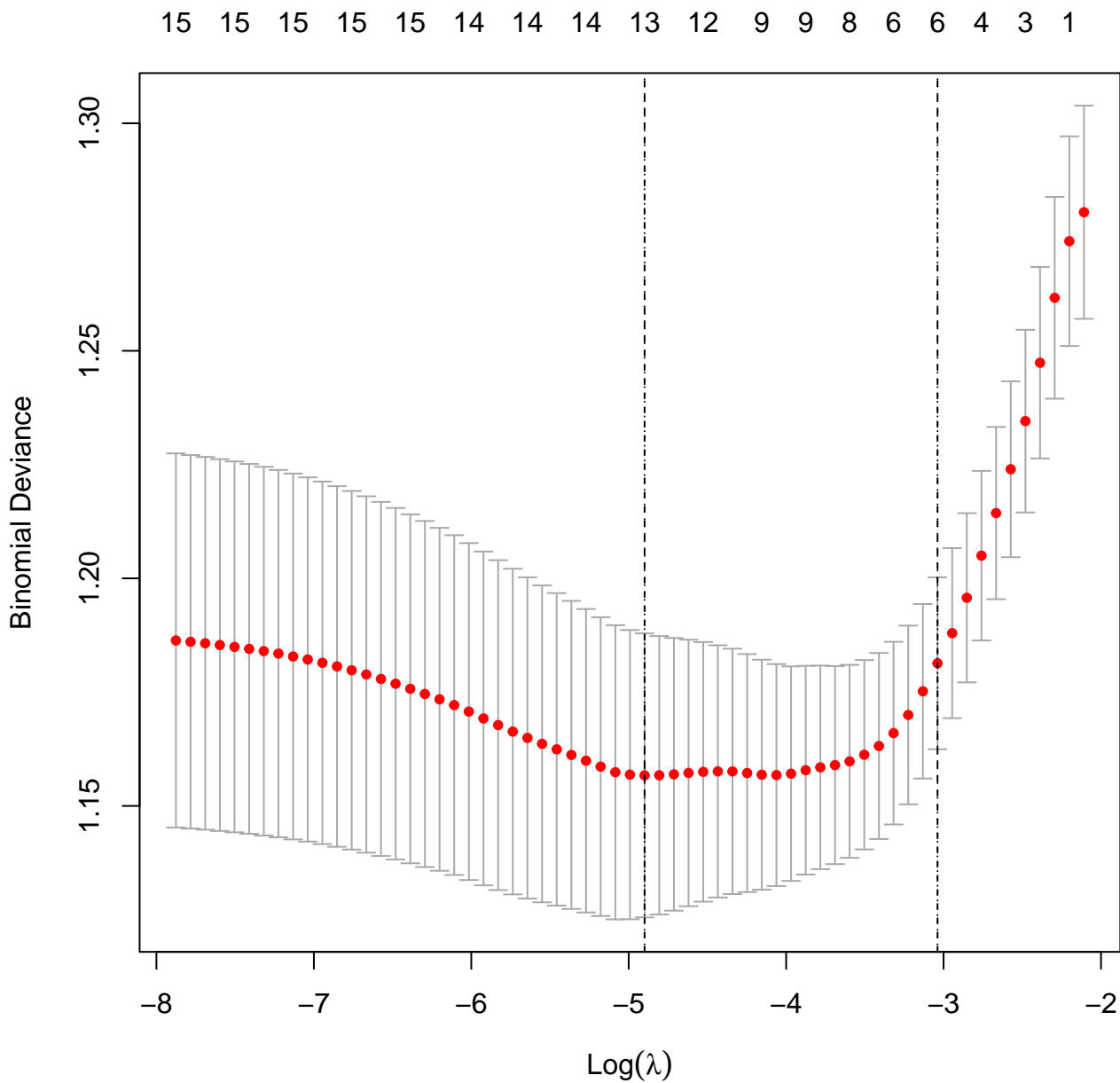

Supplement: Supplemental Information 1 [file peerj-11-15702-s006.zip › code/min.pdf]

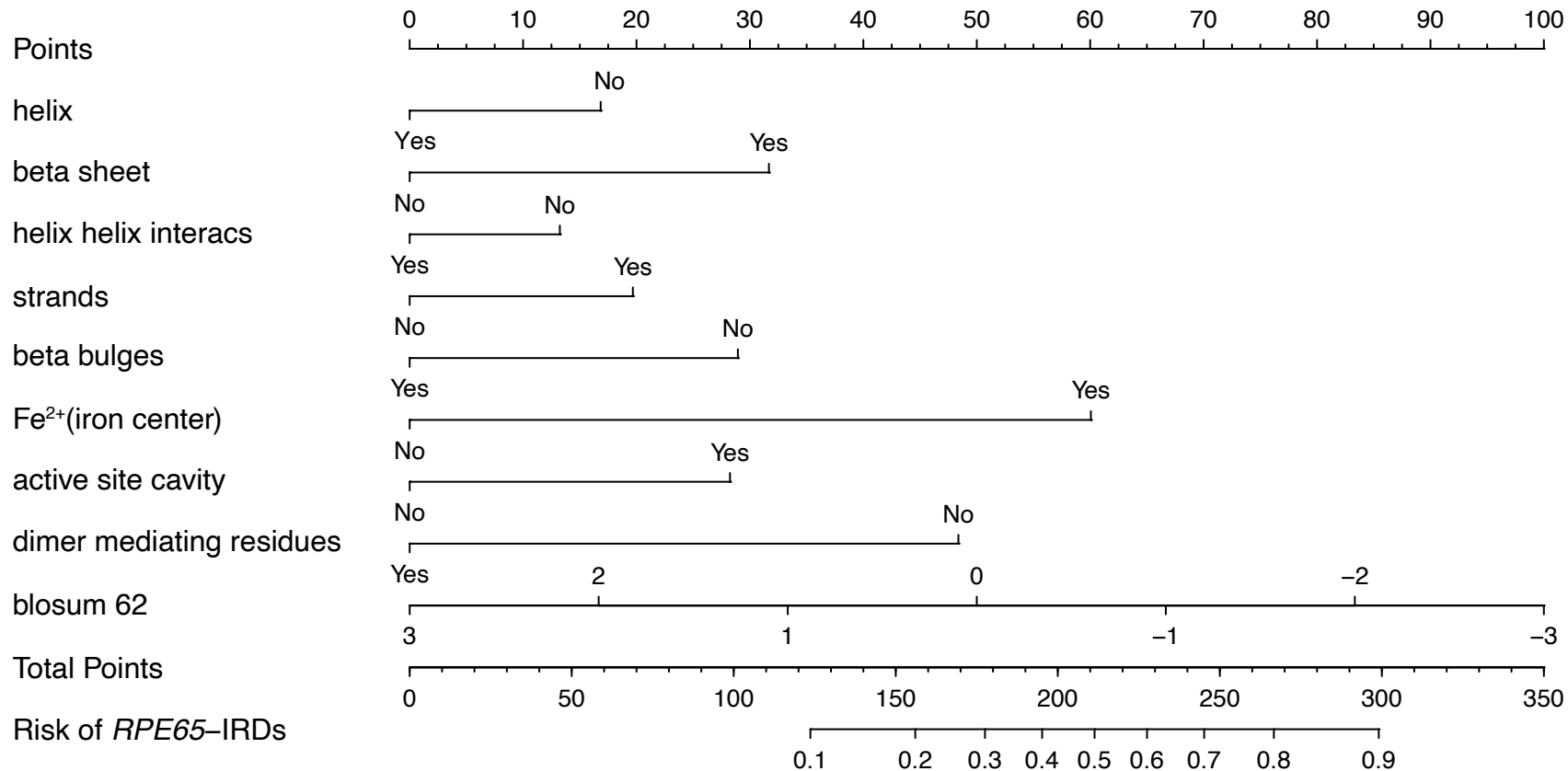

Supplement: Supplemental Information 1 [file peerj-11-15702-s006.zip › code/Nom1.pdf]

Missense-VUS

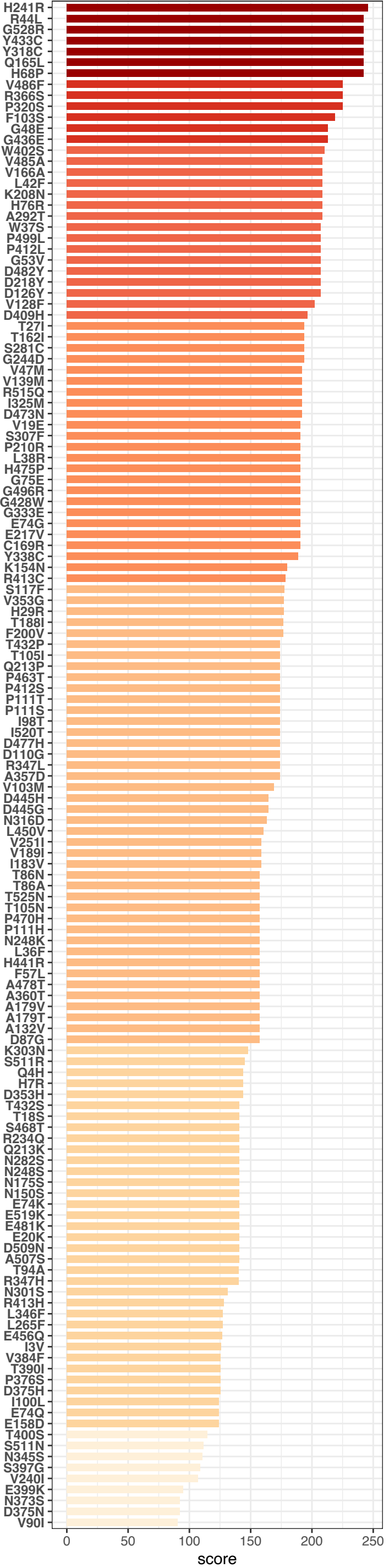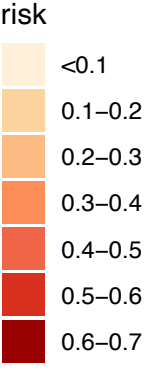

Supplement: Supplemental Information 1 [file peerj-11-15702-s006.zip › code/vus.pdf]

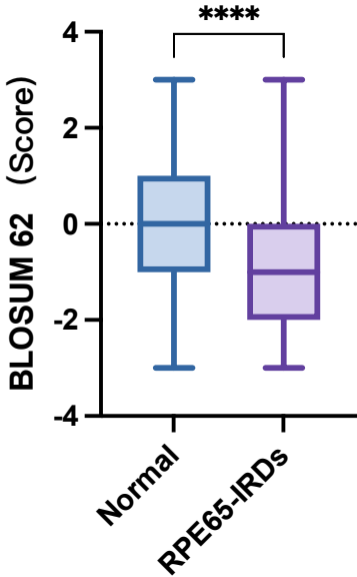

Supplement: Supplemental Information 1 [file peerj-11-15702-s006.zip › code/BLOSUM 62.pdf]

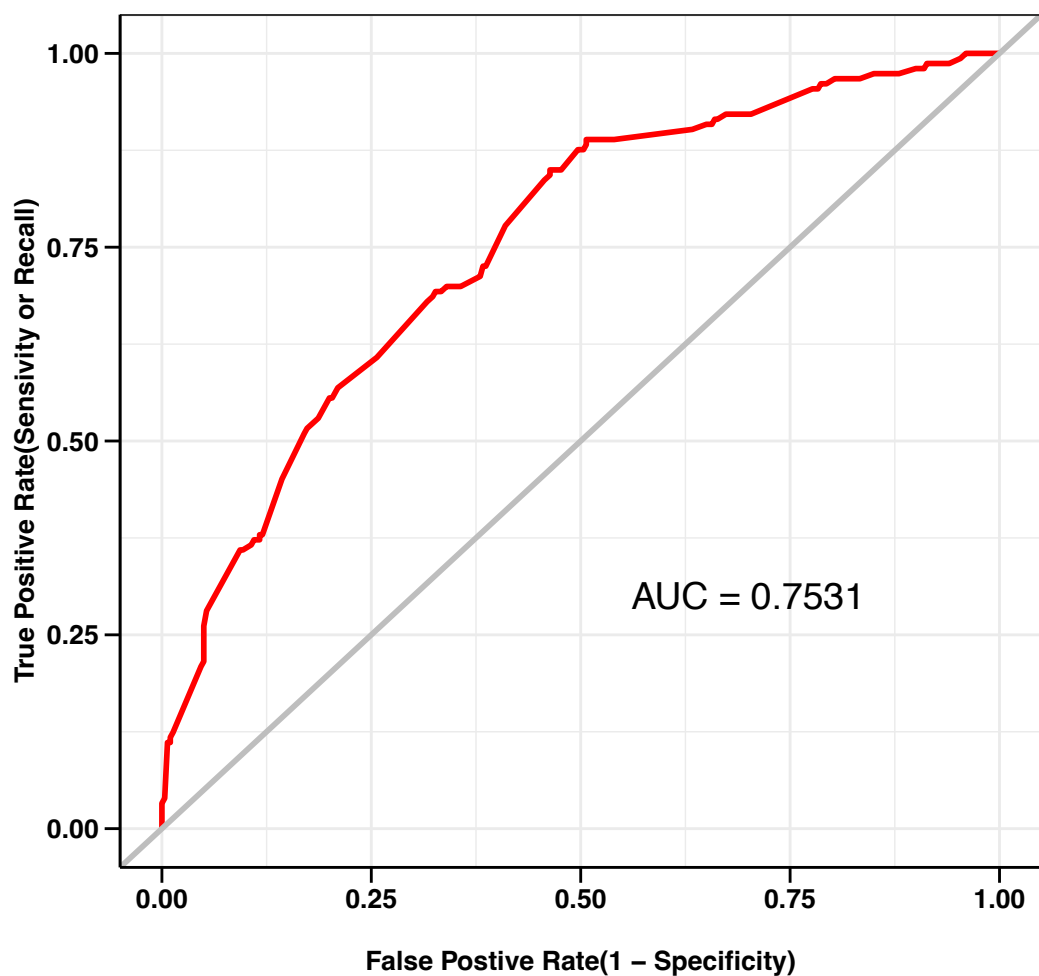

Supplement: Supplemental Information 1 [file peerj-11-15702-s006.zip › code/ROC.pdf]

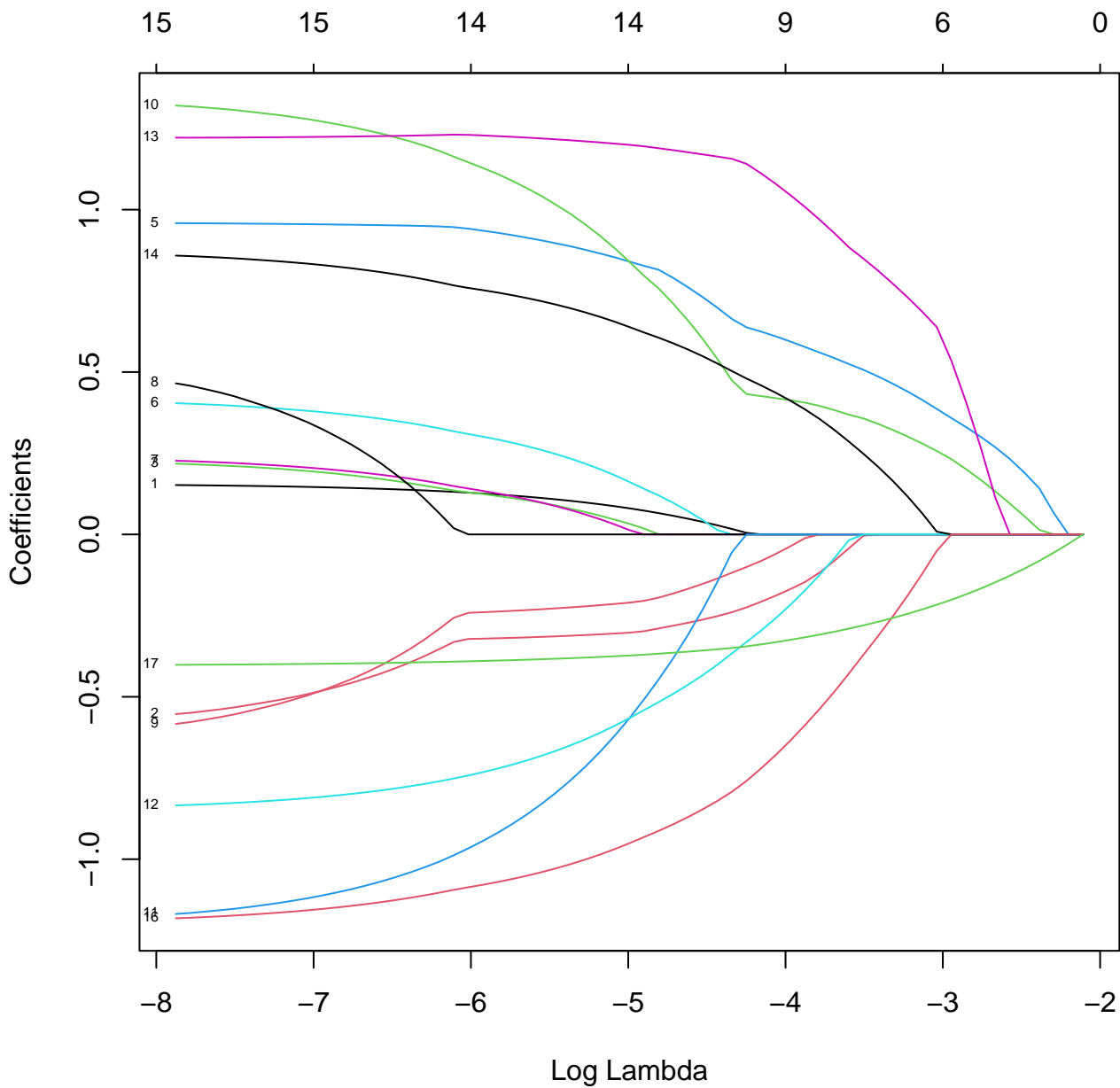

Supplement: Supplemental Information 1 [file peerj-11-15702-s006.zip › code/lambda.pdf]

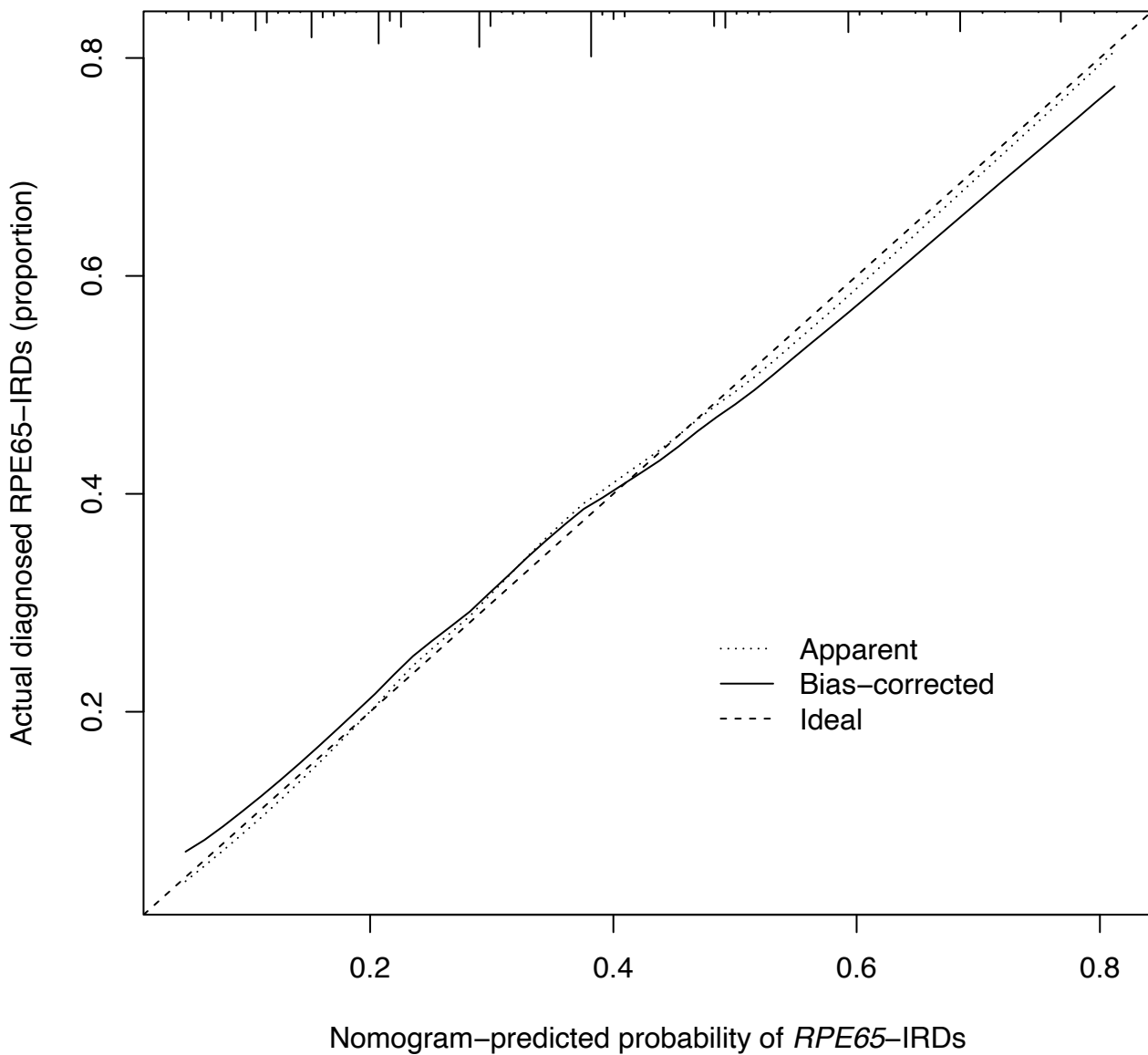

Supplement: Supplemental Information 1 [file peerj-11-15702-s006.zip › code/Calibration.pdf]
